# Polyurethane-Derived Microplastics and Nanoplastics: Sources, Human Exposure, and Toxicological and Health Effects

**DOI:** 10.3390/molecules31193517

**Published:** 2026-10-02

**Authors:** Magdalena Pietrzak, Adrian Kawecki, Magdalena Zemelka-Wiacek

**Affiliations:** Department of Clinical Immunology, Faculty of Medicine, Wroclaw Medical University, 51-616 Wroclaw, Poland

**Keywords:** polyurethane, microplastics, nanoplastics, human exposure, human biomonitoring, toxicological effects, particle–chemical mixtures, environmental aging, One Health

## Abstract

Polyurethane (PU) is one of the most widely used synthetic polymers, yet polyurethane-derived microplastics and nanoplastics (MNPs) have received less attention than particles derived from polyethylene, polypropylene, or polystyrene. This narrative review summarizes current evidence on the sources, environmental release, aging, human exposure pathways, biomonitoring findings, biological effects, and research gaps associated with PU-derived MNPs. The review synthesized 200 sources from database searches without publication-date restrictions and supplementary contextual literature, integrating environmental, experimental, biomonitoring, and clinical evidence, considering methodological limitations and publication status. PU-derived MNPs originate from diverse consumer, industrial, textile, medical, and agricultural products and undergo physicochemical transformations during environmental aging that may alter particle behavior and chemical release. Human exposure may occur through inhalation, ingestion, dermal contact, and medical procedures, while PU-containing particles have been detected in biological matrices, including respiratory samples, placenta, blood, reproductive tissues, and urine. Experimental evidence suggests that PU-derived particles and their associated leachates may induce oxidative stress, inflammation, barrier dysfunction, cytotoxicity, and immune responses, although direct evidence linking PU exposure to human disease remains limited. Integrating environmental monitoring, human biomonitoring, toxicology, and One Health approaches can improve exposure assessment, harmonize analytical methods, and clarify the ecological and clinical relevance of PU-derived MNPs.

## 1. Introduction

The extensive use of plastics and their continuous release into the environment have made microplastics (MPs) and nanoplastics (NPs) a growing concern for environmental and human health research. These particles may originate from the fragmentation of larger plastic materials or be released directly from consumer, industrial, medical, or textile products. Their properties can change after formation. Environmental aging, ultraviolet (UV) radiation, mechanical stress, hydrolysis, and chemical transformation may modify their size, morphology, surface chemistry, and ability to release or carry organic compounds [1,2,3,4,5]. As a result, MPs and NPs should be considered complex particle–chemical systems rather than inert debris.

Experimental studies relevant to human health increasingly indicate that MPs and NPs can enter human cells and disrupt mitochondrial metabolism, immune cell activity, stress responses, and inflammatory pathways. These studies also suggest that MPs and NPs may compromise biological barriers and induce oxidative stress, cytotoxicity, apoptosis, genotoxic alterations, endothelial dysfunction, and procoagulant processes [6,7,8,9,10,11,12,13]. Human biomonitoring studies have also detected plastic particles in blood, placenta, and lung tissue [14,15,16]. However, in vivo evidence remains limited, particularly for polyurethane (PU)-derived particles, because most experimental toxicology has focused on better-studied polymers such as polystyrene (PS), while the available animal evidence on PU comes mainly from aquatic and developmental models [17,18,19,20].

Polyurethane (PU) is a broad family of urethane-containing polymers formed mainly by the reaction of polyols with diisocyanates. Depending on chemical composition, additives, and degree of crosslinking, PU can occur as flexible and rigid foams, elastomers, coatings, adhesives, fibers, synthetic leather, and thermoplastic polyurethane (TPU). For this review, MPs are operationally defined as solid plastic particles with sizes ≥1 μm and <5 mm, and NPs as particles with sizes ≥1 nm and <1 μm; the term micro- and nanoplastics (MNPs) refers collectively to both fractions. NP definitions are not harmonized, with upper size boundaries of either 100 nm or 1000 nm used in the literature [21]. The broader submicrometer definition is adopted here, while retaining the particle-size ranges reported in individual studies. These classification boundaries are independent of the size ranges accessible to the analytical methods used. PU is relevant to MNP research because it combines high-volume use with substantial material diversity: different PU formulations may differ in physicochemical properties, persistence, biodegradation, fragmentation behavior, and capacity for chemical release [22]. Although PU is produced in lower volumes than major commodity polymers such as polypropylene (PP), polyethylene (PE), or polyvinyl chloride (PVC), it remains a major polymer group. In 2023, PU accounted for approximately 5.3% of global plastics production, corresponding to about 21.9 million tons, compared with 19.0% for PP, 14.0% for low-density and linear low-density polyethylene (PE-LD/LLD), 12.8% for PVC, and 12.2% for high- and medium-density polyethylene (PE-HD/MD) [23]. In China, PU production was 11-fold higher in 2022 than in 2000, in-use stocks reached 9.09 × 10^10^ kg, construction accounted for more than 30% of stocks, and textiles, apparel, and footwear generated 41.4% of PU waste [24]. These applications create multiple opportunities for PU-containing materials to wear, fragment, and release MPs and NPs during production, use, washing, abrasion, weathering, and waste handling [25,26,27,28,29,30,31,32]. PU-containing products therefore represent potential sources of MPs and NPs [25,26,27,28,29,30,31,32]. Although PU is less abundant than dominant commodity polymers in global production statistics, PU-derived MNPs may still contribute to environmental release, human exposure, particle aging, chemical release, and biological effects.

**Figure 1:**
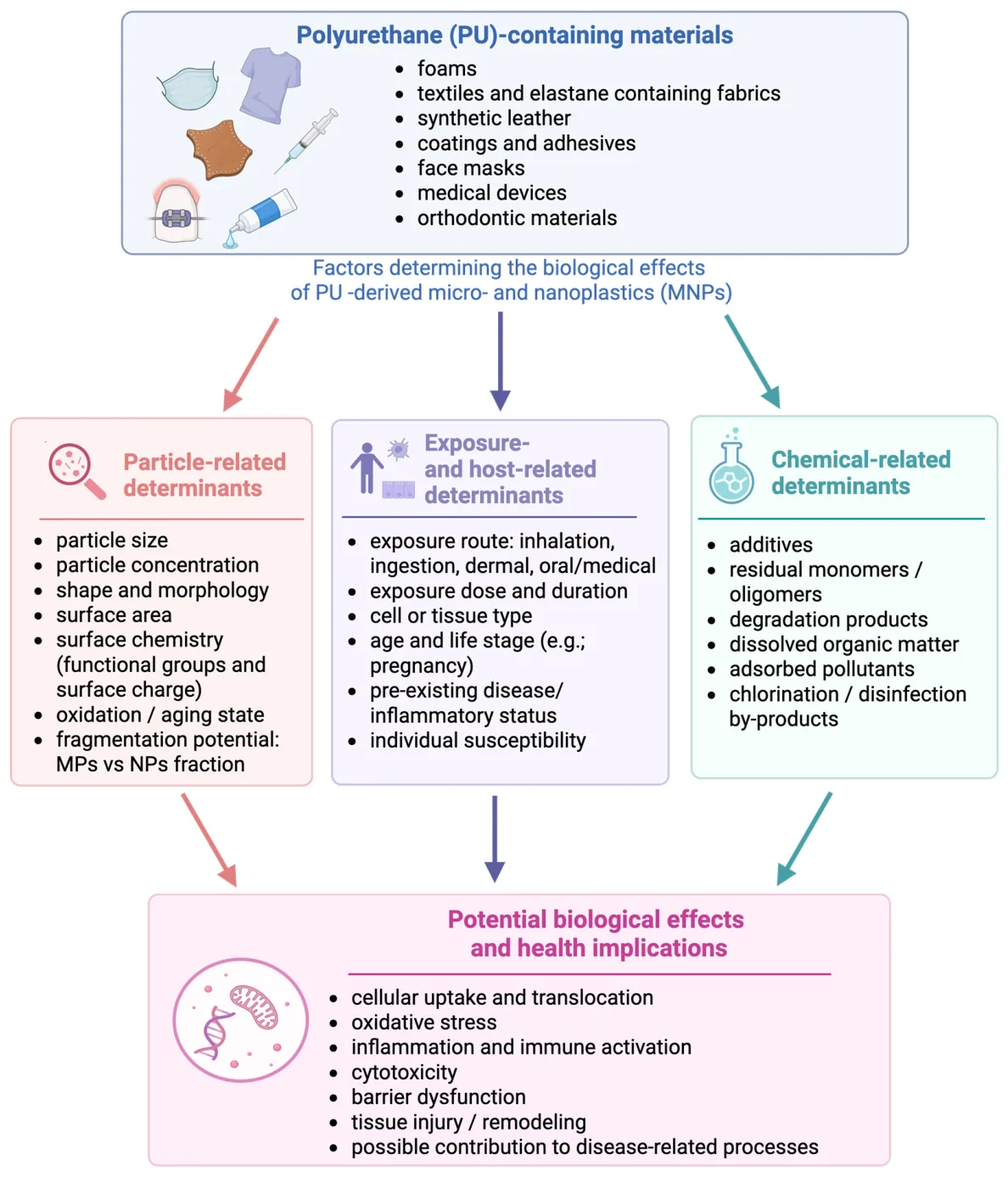
Factors determining the biological effects of PU-derived MNPs. The diagram illustrates the main particle, chemical, host and exposure factors that may influence biological responses to PU-derived MNPs. Arrows indicate conceptual links between PU-containing materials, key determinants, and potential biological effects. Abbreviations: MNPs = micro- and nanoplastics; MPs = microplastics; NPs = nanoplastics; PU = polyurethane.

PU-derived MPs and NPs should also be considered within a broader One Health and exposome perspective. Their relevance is not limited to a single exposure route but reflects the entire life cycle of PU-containing materials, from production and use to environmental release, ecosystem contamination, food-chain transfer, and human exposure. During this process, PU particles may interact with additives, degradation products, dissolved organic matter (DOM), and adsorbed pollutants, forming dynamic particle–chemical mixtures that can change during aging and transport [33,34,35,36]. This perspective is important because the biological relevance of PU-derived particles is determined not only by particle properties but also by associated chemicals, exposure conditions, target biological interfaces, and host susceptibility factors, as summarized in Figure 1.

Recent reviews have synthesized evidence on the environmental distribution, human exposure, and potential health effects of microplastics and nanoplastics [8,37], while a rapid systematic review evaluated evidence concerning digestive, reproductive, and respiratory outcomes [38]. These syntheses provide a broader health context for examining individual polymer families. For PU, differences in backbone chemistry, crosslinking, and formulation influence degradation and the composition of released chemicals [22,33], supporting a closer examination of material-specific exposure and biological findings. The present review complements these broader assessments by connecting PU-containing products, including occupational and medical sources, with particle release, human biomonitoring, experimental effects, and analytical challenges. Examining PU separately helps distinguish findings concerning its particles and leachates from evidence obtained with other polymers or mixed-polymer samples, thereby clarifying both the available PU-specific evidence and the limits of current health-related conclusions.

This review aims to summarize current evidence on PU-derived MPs and NPs as an understudied source of environmental contamination and human exposure. It discusses PU sources, degradation and chemical release, exposure pathways, human biomonitoring, biological effects, analytical limitations, and future research needs. Particular attention is given to the gap between particle detection and clinically meaningful risk assessment.

**Figure 2:**
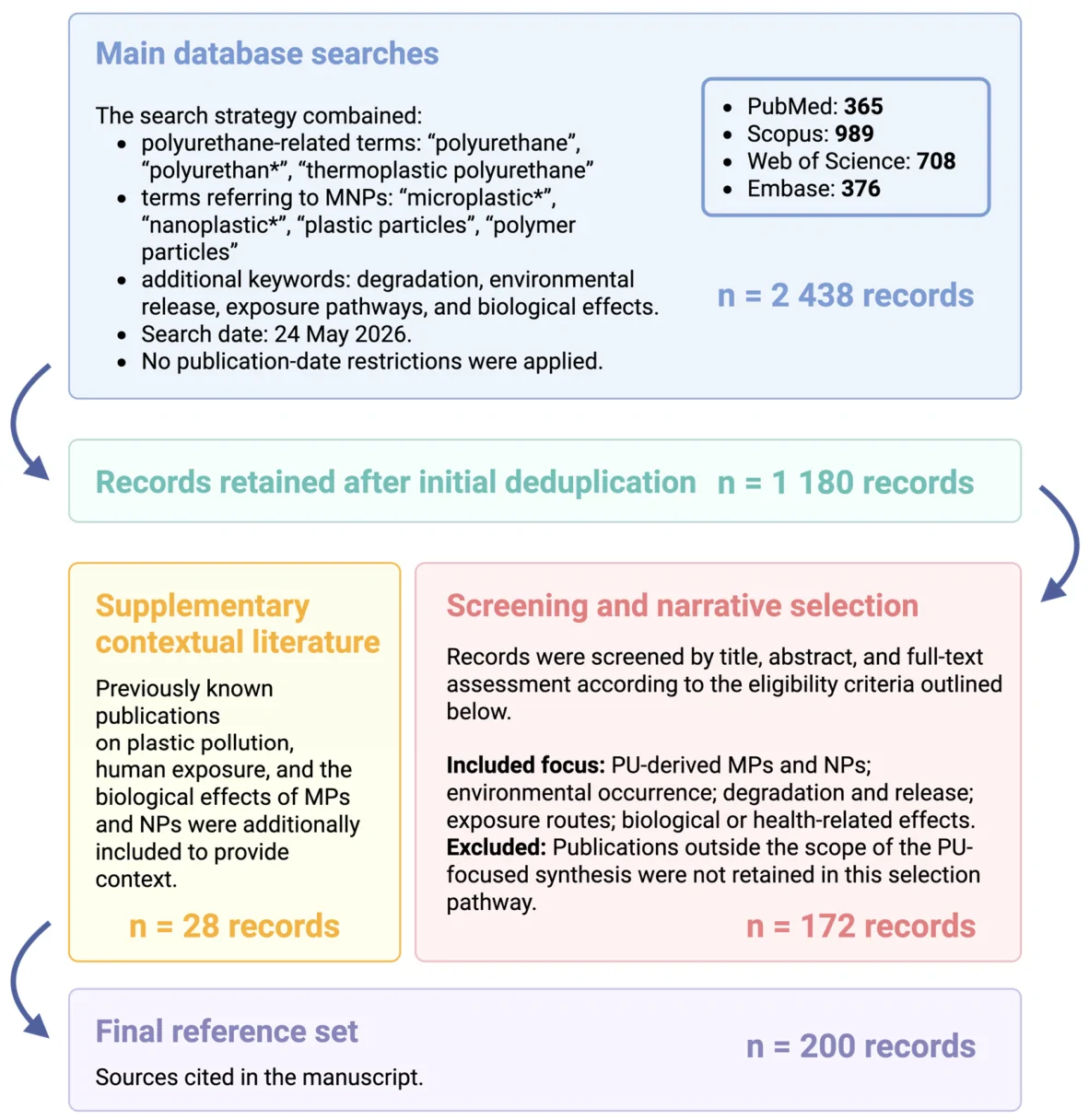
Literature search and narrative study-selection process. The diagram outlines the database searches, screening, eligibility assessment, and final selection of sources. Arrows indicate the study-selection flow; asterisks (*) denote truncation wildcards in the search terms. Abbreviations: MNPs = micro- and nanoplastics; MPs = microplastics; NPs = nanoplastics; n = number of records; PU = polyurethane.

The literature search for this narrative review was conducted in PubMed/MEDLINE, Scopus, Web of Science Core Collection, and Embase using the following core query, adapted to each database: (polyurethane OR polyurethan* OR “thermoplastic polyurethane”) AND (microplastic* OR nanoplastic* OR micro-plastic* OR nano-plastic* OR “plastic particle” OR “plastic particles” OR “polymer particle” OR “polymer particles”). Records were initially screened by title and abstract, followed by full-text assessment of their relevance to the scope of this review. Publications already known to the authors were also selected to provide broader context on plastic pollution, human exposure, and biological effects. The search date, eligibility criteria, and numbers of records retained through both selection pathways are presented in Figure 2. Study characteristics and findings were extracted for tabular comparison. Evidence was considered qualitatively in light of publication status and reported analytical characteristics.

## 2. PU as a Source of MNPs

The release of PU-derived MNPs is strongly dependent on the product. Crosslinking density and hard-segment content can influence the biodegradation of PU microplastics, whereas product structure, including coatings, textile layers, elastomeric films, foams, and medical components, determines which physical and chemical stressors affect the material during use and disposal [22,24,25,26,30,31,32]. In practice, PU-containing fabrics, synthetic leather, TPU films (thin, flexible thermoplastic polyurethane sheets), face masks, orthodontic materials, and disposable medical devices may be exposed to UV radiation, abrasion, washing, disinfection, or biological fluids such as saliva and blood; these conditions may promote particle release, surface transformation, or chemical leaching [23,25,26,27,28,29,30,31,32,39,40,41]. Therefore, PU-derived MNPs should be evaluated in relation to both material formulation and product use, because these factors influence particle number, size, morphology, surface oxidation, associated chemicals, and environmental redistribution [24,25,26,27,28,33,39,40,41,42].

### 2.1. Material Diversity and Major Applications of PU

PU denotes a heterogeneous group of urethane-containing polymers whose properties depend on backbone chemistry, soft and hard segments, crosslinking, additives, and formulation. This chemical and structural variability allows PU to be used as flexible and rigid foams, elastomers, TPU, coatings, films, fibers, synthetic leather, composites, adhesives, sealants, and selected medical or dental materials. These material categories do not necessarily predict environmental behavior on their own. In comparative experiments with PU and TPU, increasing crosslinking density and hard-segment content reduced biodegradation, while a hydrolysis stabilizer reduced fragmentation without affecting the conversion of polymer carbon to CO_2_ [22]. Product structure also matters: PU-coated textiles and synthetic leather can release both coating-derived material and substrate fibers [25,26], whereas UV-aged TPU powders showed crosslinking and agglomeration alongside the release of smaller fragments [40]. Thus, environmental behavior depends on both formulation and product structure, rather than on the PU designation alone.

PU-derived MNPs are mainly formed as secondary particles, meaning fragments generated from larger PU-containing products during use, abrasion, aging, cleaning, weathering, or disposal. Because PU is widely used in construction, insulation, furniture, mattresses, textiles, footwear, synthetic leather, coatings, adhesives, sealants, masks, consumer goods, and selected medical or dental devices, its potential release pathways are strongly linked to product use and waste handling [24,25,26,27,28,29,30,31,32]. Among these applications, construction materials and textile-, apparel-, and footwear-related products are particularly important because they represent major PU reservoirs and waste streams [24]. In addition, a Swiss probabilistic emissions model combined data on polymer use, product categories, end-of-life pathways, and release scenarios to estimate releases of five commodity plastics to the environment. In 2018, PU ranked second among the analyzed polymers, with an estimated 179 ± 98 tons of macroplastic and MP emissions to the Swiss environment. Only polyamide (PA) had higher estimated emissions, at 208 ± 76 tons, whereas polycarbonate (PC), poly(methyl methacrylate) (PMMA), and acrylonitrile butadiene styrene (ABS) were lower, at 79 ± 26, 36 ± 23, and 25 ± 6 tons, respectively [42]. Different PU-containing products follow different use and degradation scenarios, which determine when particles are released and which exposure route may become relevant. PU-coated textiles, synthetic leather, TPU films, and masks may release particles during friction, washing, UV exposure, photoaging, or disinfection, leading mainly to environmental transfer, wastewater contamination, dermal contact, or inhalation of airborne particles [25,26,27,28]. Orthodontic materials are exposed to saliva and mechanical wear in the oral cavity, whereas selected medical devices may release particles during contact with infusion fluids, extracorporeal circulation, hemodialysis, or interventional procedures [29,30,31,32]. Therefore, exposure to PU-derived MNPs should be interpreted in relation to the original product, degradation conditions, and route of contact.

### 2.2. Formation and Environmental Release Pathways of PU-MNPs

The formation and release of PU-derived MNPs depend on the stressors acting on the original material and on the environmental compartment into which the particles are released. Experiments with thin TPU films (flexible thermoplastic PU sheets used as model materials) show how combined UV radiation and mechanical abrasion can accelerate surface degradation and increase MP release [39]. Outdoor studies involving trail running and hiking further indicate that particles from footwear and clothing can be transferred from PU-containing products to surrounding environments [43,44,45]. In composite products, such as PU-coated textiles and synthetic leather, weathering may affect both the PU coating and the underlying textile substrate, resulting in the release of fibers, coating fragments, additives, and aged surface material [25,26]. Accordingly, product design and use conditions determine the composition of the released fraction before its subsequent transport through environmental pathways.

After release, PU-containing particles can be transported through air, dust, stormwater runoff, wastewater, sludge, soils, and aquatic systems. Evidence from built and industrial environments illustrates this transport and shows that PU abundance relative to other polymers varies between settings. PU, PVC, and PE were among the dominant polymers in Vienna road dust [46]. In stormwater runoff from university parking lots and roadways, PU accounted for 11% of identified MPs, compared with 7% for PVC [47]. In treated effluents from six dyeing and printing wastewater treatment plants, PU represented an average of 16.8% of detected MPs, compared with 57.5% for PE [48]. Wastewater can also receive synthetic fibers from consumer materials, including non-PU sources such as wet wipes, showing that different polymer streams may converge in the same treatment systems [49]. These release and transport pathways represent only the first stage of the environmental trajectory of PU-derived particles. Subsequent aging, oxidation, sorption, and chemical release may further modify these particles after their entry into environmental compartments.

## 3. Degradation, Aging and Chemical Release

After detachment from a product, PU particles are exposed to light, water, mechanical stress, oxidants, and biological activity, all of which may gradually modify their surface and the chemicals associated with them [25,26,33,34,35,39,40,41,50]. Therefore, environmentally aged PU particles may differ substantially from the material originally released, in both their physical properties and the chemicals they carry or release.

### 3.1. Environmental Weathering, Fragmentation and Photoaging

After release, PU- and TPU-derived particles undergo physical and chemical changes that should be distinguished. Fragmentation describes the physical breakup of a material into smaller particles, whereas chemical degradation involves changes in polymer molecular structure, such as chain scission through photo-oxidation or hydrolysis. Chemical changes may facilitate fragmentation, but increased particle release during washing or abrasion does not by itself demonstrate polymer-chain degradation. Particle counts and size distributions should therefore be interpreted alongside measurements of molecular weight, functional groups, and dissolved degradation products [39,40,41,51]. PU-containing masks provide an example of how mechanical wear during use, washing, disinfection, and environmental aging can jointly promote particle release. New PU-containing masks released 163 ± 83 MPs per mask, whereas used masks released 607–2167 MPs per mask; washing increased release to as many as 30,708 MPs per mask, and photoaging of PU-containing layers generated approximately 1.0 × 10^5^ MPs and 3.5 × 10^12^ NPs per layer [27]. Laundering and disinfection of reusable masks may further contribute to both particle release and chemical emission from polymer-containing layers [28,52].

PU-coated fabrics and synthetic leather show that particle release may occur together with chemical transformation. During photodegradation of polyester fabrics with a PU coating, the coated material weakened and released both micro- and nanoscale particles, with particle generation after 360 h reaching 9.32 × 10^7^ MPs/g and 2.70 × 10^11^ NPs/g [25]. In PU synthetic leather, the coating initially delayed fiber release, but it also underwent more pronounced oxidation and generated chemically complex leachates during photoaging [26]. Thermal and thermo-oxidative processes are mainly relevant to waste handling or uncontrolled burning, where PU-containing materials may undergo chemical transformation rather than simple particle fragmentation [53,54]. PU persistence also depends on formulation, because biodegradation is influenced by backbone structure, ester or ether linkages, hard-segment content, crosslinking density, and additives. Partial biodegradation may compromise the integrity of PU materials without fully mineralizing the polymer, leading to smaller fragments, soluble intermediates, or microbially colonized particles [22,55,56,57,58,59,60,61].

The measurable consequences of aging depend on both exposure conditions and PU formulation. In PU-coated polyethylene terephthalate (PET) fabrics, changes in surface morphology, molecular weight, and crystallinity were examined alongside particle release, linking physical deterioration with molecular and structural changes in the composite material [25]. In a study of TPU powders, UV aging induced crosslinking and gel formation, while microscopy indicated fragment release through surface ablation. The measured size of the aged TPU particles increased, which the authors attributed to softening and crosslinking-driven agglomeration, despite simultaneous release of smaller fragments [40]. Thus, aging does not necessarily produce a uniform decrease in particle size or molecular weight. Dissolved organic matter provides a complementary measure of material transformation, although its release may reflect both polymer degradation and leaching of pre-existing constituents [33,35,41]. These findings support assessing particle-size distributions, surface morphology, and chemical changes together.

### 3.2. Leaching of Additives and Degradation Products

PU-derived MPs and NPs may release chemicals already present in the material, as well as compounds formed during aging. This release can involve diffusion of additives, residual monomers, oligomers or chain-extender-derived compounds, oxidative bond cleavage, photodissolution, and reactions occurring during water treatment or disinfection [25,26,28,33,35,50,62,63]. UV-aged TPU can become more reactive during chlorination than pristine TPU, with more pronounced surface transformation, a 1.6-fold increase in released organic compounds, and formation of disinfection by-products [50]. Pre-ozonation before chlorination may further increase disinfection by-product formation from MPs, while fluorescent additives can also leach from plastic particles into aquatic media [63,64]. In addition to releasing their own additives and degradation products, aged PU and TPU particles may also act as carriers of environmental contaminants. UV aging can change particle morphology and surface oxidation, which may alter how strongly particles bind pollutants such as bisphenols, 17α-ethinyl estradiol, and polycyclic aromatic hydrocarbons, as well as how easily these compounds can later be released from the particle surface [65,66,67]. Therefore, exposure to aged PU particles may include not only the particles themselves but also a chemical fraction consisting of leached additives, degradation products, and environmental contaminants carried on the particle surface [33,34,50,63,65,66,67].

## 4. Human Exposure Pathways

**Figure 3:**
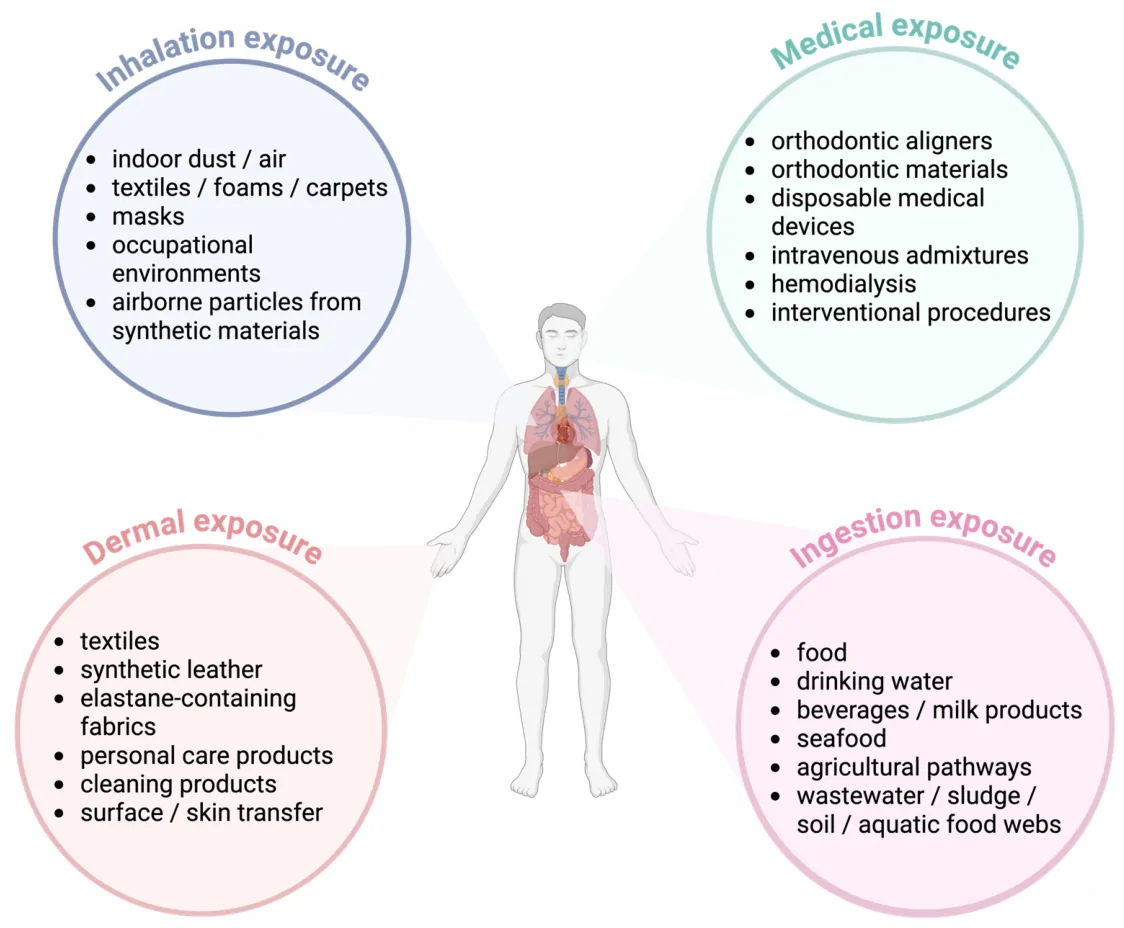
Human exposure pathways for PU-derived MNPs. Abbreviations: MNPs = micro- and nanoplastics; PU = polyurethane.

PU-containing materials are present in indoor environments, consumer products, food- and water-related systems, and selected oral or medical applications [25,26,27,28,29,30,31,32,68,69,70]. The route of exposure depends on the release setting and transfer pathway: particles may be inhaled, ingested, transferred through contact with skin, or introduced into the body via fluids or blood products administered during medical procedures (Figure 3) [29,30,31,32,68,69,70,71,72].

### 4.1. Inhalation Exposure

Inhalation is a plausible route of exposure to PU-containing MPs because PU is present in materials that can contribute to indoor dust and airborne particles, including foams, carpets, textiles, coatings, flooring, masks, and other synthetic materials. Indoor dust is especially relevant because it accumulates particles released from household products and can be inhaled or ingested after resuspension or hand-to-mouth transfer. In an international survey of dust from 108 homes in 29 countries, PU was the dominant polymer among MP fibers in samples from low-income countries, with particularly high modeled infant exposure through inhalation and ingestion [68]. Source-apportionment analysis from 39 Chinese cities further linked indoor MPs to textile release, attributing 43–59% of indoor MPs to textiles and 98% of detected PU particles to textile-related sources [73]. Face masks provide another example of a source close to the body because selected components such as straps, ear loops, or accessory layers may release PU-related particles close to the breathing zone [70]. These exposure pathways are relevant to topics addressed in recent European Academy of Allergy and Clinical Immunology (EAACI) environmental-science guidelines, which discuss microplastics as emerging environmental pollutants that may contribute to epithelial barrier disruption, oxidative stress, inflammatory responses, and asthma-related outcomes [74,75].

Airborne MPs have also been reported in outdoor air, roadside and residential areas, urban environments, remote locations, and atmospheric deposition samples [76,77,78,79,80,81,82,83]. PU was identified among the detected polymers in selected studies, indicating that PU-containing particles may contribute to inhalation exposure beyond indoor environments [76,77,78,79,80,81,82,83,84]. PU-containing particles have been reported in sputum and bronchoalveolar lavage fluid (BALF), further supporting inhalation as a plausible exposure route, while material identified as polyurethane glycol has also been detected in middle-ear effusion fluid, for which the route of exposure remains uncertain [85,86,87,88,89,90,91].

Occupational and activity-related environments may increase inhalation exposure when activities involving synthetic materials generate particles in poorly ventilated spaces. Nail salons provide one documented example because cosmetic procedures involve polymer-containing materials, filing, abrasion, and close-range exposure. In this environment, the mean concentration of total airborne MPs was 46 ± 55 particles/m^3^, and estimated annual inhalation exposure to total MPs was 67,567 ± 81,782 particles/year. PU accounted for 13% of identified polymers, providing information on mixture composition rather than a directly quantified PU-specific inhalation dose [92]. Environmental setting may also influence the type and amount of MPs detected in the lower respiratory tract. In BALF samples, differences in microplastic profiles were associated with proximity to roads, coastlines, and industrial areas, suggesting that local environmental sources may shape inhalation exposure [86]. Wastewater-treatment environments represent another airborne source category, because MPs have been detected in air from wastewater treatment plants and may carry potentially pathogenic bacteria [93]. Textile-related sources may contribute to several environmental compartments. Textile wastewater contains MPs [94], while elastane-containing and household textiles can release synthetic microfibers during ordinary use and laundering [95,96]. Because elastane, also known as spandex, is a polyurethane-based fiber, these materials may contribute to PU-containing fibers in dust, wastewater, and airborne particles, linking textile use with both environmental release and potential inhalation exposure [95,96].

### 4.2. Gastrointestinal Exposure

Gastrointestinal exposure to PU-containing MPs and NPs may occur when particles are ingested with food, drinking water, or other beverages, or following transfer from contaminated hands and surfaces. Contamination can arise before consumption, including during production, processing, packaging, transport, storage, or preparation of food and water products [69,97,98].

Seafood provides one of the clearest links between environmental contamination and diet, because aquatic organisms can ingest particles from contaminated water, sediment, or feed and may later be consumed by humans. PU has been identified among polymers detected in selected studies of aquatic food sources, including farmed oysters, commercial finfish and shellfish, epipelagic prawns, seafood from the Persian Gulf, burbot tissues, and oysters from the Mississippi Sound [99,100,101,102,103,104,105,106]. Oysters are particularly useful as an example because they are filter feeders. In oysters from the Mississippi Sound, total putative MP concentrations ranged from 4.7 to 30.7 particles/g wet weight, with PU identified as one component of the polymer mixture [106]. Data from farmed oysters, fish, shellfish, prawns, crabs, and edible fish tissues further indicate that aquatic food systems may contribute to dietary exposure, although actual intake depends on the tissue consumed, preparation method, and individual diet [99,100,101,103,104,105,107]. PU-containing MPs have also been reported in selected matrices relevant to ingestion, including beverages, milk and milk-based products, commercial salts, infant formula, and breast milk [108,109,110,111,112,113,114]. The detection of PU among MPs in breast milk and infant formula also indicates a potential source of early-life dietary exposure [108]. Freshly prepared milk tea and coffee samples from China contained MPs in 93% of beverages, with PA, PU, and PET as the main polymers. The estimated adult intake of 24.60 particles/year/kg body weight referred to total MPs rather than PU alone [111]. Together, these studies indicate that PU may occur not only in seafood and drinking water but also in commonly consumed beverages and milk-based products, although PU-specific dietary intake remains difficult to estimate [109,111,113,114].

Drinking water represents a repeated daily exposure route. PU or TPU has been identified in several drinking-water-related systems, including bottled water, tap water, groundwater, freshwater, long-distance raw-water pipelines, and drinking-water treatment plants [69,84,115,116,117,118,119,120,121]. This indicates that PU-related particles may reach water intended for consumption at different stages, from source waters and treatment processes to distribution pipelines and packaging. Fluorophore-based screening of bottled and environmental waters should be interpreted as complementary methodological evidence, as it can indicate particle presence but provides less precise polymer identification than spectroscopic analysis [119]. Spectroscopic studies nevertheless show that particles may persist across water systems. Laser direct infrared spectroscopy (LDIR)-based analysis of bottled water reported an average total MP concentration of 96 particles/L, with PET and/or PU among the abundant polymers [84]. In drinking-water treatment plants and bottled water from Jordan, MPs were detected in all raw-water samples, and treatment reduced but did not eliminate particles, with PU among the main polymers [120]. Water-treatment processes, including chlorination, ozonation, UV exposure, and combined oxidation–chlorination, may further transform PU and TPU particles by modifying their surfaces, promoting organic release, altering adsorption behavior, and contributing to disinfection by-product formation [50,64,122,123,124].

Indirect pathways connect environmental contamination with later ingestion through food and water systems. PU-containing or PU-relevant MPs may enter soils, sediments, rivers, wastewater systems, landfill leachates, agricultural fields, and aquatic food webs, where they can be redistributed before reaching drinking water, crops, feed, or consumed organisms [48,115,125,126,127,128,129,130,131,132,133,134]. Wastewater treatment plants remove part of the particle load, but treated effluents and sludge may still transfer MPs to receiving waters and agricultural soils [48,128,129]. Agricultural soils can receive MPs from plastic mulching, biosolids, polymer-coated fertilizers, textile-derived particles, and atmospheric deposition [125,131,134,135,136,137,138,139,140,141]. Controlled-release fertilizers with PU or other polymer coatings, as well as commercial aquaculture feed, provide further examples of how PU-related particles may enter food production systems [135,137,138,139,141].

### 4.3. Dermal Exposure

Dermal exposure to PU-containing MPs is less well documented than inhalation or ingestion, but it remains relevant because many PU-containing materials are used in direct contact with the skin. These include textiles, elastane-containing fabrics, coatings, synthetic leather, household fabrics, and other flexible polymer-containing materials [25,26].

Elastane-containing fabrics are important in this context because elastane is a PU-based fiber. Laundering experiments show that microfiber release can increase with elastane content, and household textiles may release synthetic microfibers during ordinary use and washing [95,96]. Released fibers from PU-containing textiles may therefore remain in contact with the skin, but they may also be transferred to dust, air, surfaces, hands, or wastewater, linking dermal contact with secondary inhalation, ingestion, and environmental exposure.

Consumer products create additional contact and transfer pathways. Facial care products, scrubs, wet wipes, cleaning products, and other polymer-containing materials can release MPs during application, abrasion, rinsing, or disposal [49,142,143,144]. Facial cleansers, scrubs, and other rinse-off cosmetics represent a relevant contact pathway because they are applied directly to the skin and then washed into wastewater. PU has been detected in some facial care products, although intentionally added exfoliating or abrasive polymer particles used in scrubs and similar personal care products are more often composed of PE, PP, or PS [142,144].

### 4.4. Medical Exposure

Medical exposure differs from environmental inhalation or ingestion because polymer-containing materials may be placed directly in contact with saliva, oral mucosa, infused fluids, blood, or extracorporeal circulation systems.

Medical devices are most relevant to PU exposure when PU-containing components are used to administer blood products or infusion fluids, or in extracorporeal circulation or other medical procedures. MPs have been reported in intravenous admixtures and during hemodialysis, providing evidence of exposure to mixed polymers in medical settings [32,71]. Following percutaneous coronary intervention (PCI), total blood MP concentrations increased, with PU among the identified polymers; this overall increase should be distinguished from a PU-specific exposure estimate [31]. In the PCI study involving 23 patients, total MP concentrations increased from 4.96 ± 3.40 to 93.57 ± 35.95 particles/10 mL of blood, and the maximum observed particle diameter increased from 50 to 213 μm [31]. In nine hemodialysis patients, blood MP counts increased after treatment, with detected particles predominantly measuring 20–50 μm [32]. Among 11 intravenous admixtures examined, 99% of detected MPs measured less than 100 μm [71]. These numerical findings concern the overall polymer mixtures and suggest that interventional and extracorporeal procedures may introduce particles directly into the circulation, bypassing the external barriers involved in inhalation, ingestion, or dermal contact.

Orthodontic materials provide a direct oral exposure setting because aligners and urethane-based printed appliances remain in contact with saliva and are repeatedly subjected to insertion and removal, chewing forces, and mechanical wear. A widely used example is Invisalign, whose SmartTrack material is described as a multilayer thermoplastic polyurethane/co-polyester material designed for controlled tooth movement [145,146]. Exposure of orthodontic splints to artificial saliva has also been shown to promote the release of secondary MPs, including from PU-based materials [29]. In a laboratory study using artificial saliva and daily agitation for one week, blank-corrected mean flow cytometry counts were 2559 events for PU-based Zendura and 7403 for multilayer PU-containing Invisalign in the analyzed 500 μL aliquots. Electron microscopy showed particle diameters spanning approximately 10 nm–100 μm across the tested formulations [72].

Together, these findings support further investigation of the clinical relevance of device-related particle release, with attention to exposure route and PU-specific measurements.

### 4.5. Internal Exposure

PU has been identified in human biological samples, predominantly as micrometer-sized particles or through polymer mass measurements without particle-size resolution [31,85,86,87,88,89,90,91,108,147,148,149,150,151,152,153,154,155,156,157,158,159,160]. The original sources, timing, and routes of exposure often remain uncertain. These biomonitoring findings and their analytical limitations are discussed in Section 5.1.

## 5. Evidence of Biological and Health-Related Effects

The biological and health-related relevance of PU-derived MNPs remains difficult to define because PU-specific evidence is still limited and methodologically heterogeneous. In contrast to the broader toxicological literature on MPs, few in vivo studies have focused on PU, and most have used aquatic models [17,18,19,20].

The evidence discussed below is interpreted according to the tested exposure form: PU/TPU particles, PU-derived DOM or leachates, urethane-containing materials, and samples containing multiple polymers. Biological responses to DOM or leachates are distinguished from responses to particle exposure. Findings for urethane-containing formulations are considered material-specific, while effects or associations reported for mixed-polymer samples are not attributed to PU unless polymer-specific results are available. Even in PU/TPU particle studies, the respective contributions of particles and released chemicals cannot be resolved without appropriate controls.

### 5.1. Detection of PU in Human Biological Samples

Samples collected during pregnancy and early life are important because they may reflect exposure during sensitive developmental periods. PU has been reported among the polymers detected in placentas and meconium, while infant feces, breast milk, and infant formula were also positive [108,147]. In these matrices, most detected particles were in the micrometer range, with 20–50 μm particles accounting for more than 74–76% of MPs [108]. An earlier clinical study also detected PU in placental tissue [148]. However, because PU was additionally found in airborne fallout from the operating room, this study highlights the need for rigorous contamination-control procedures and cautious interpretation of positive MNP biomonitoring findings [148]. Additional studies reported MPs, including PU, in maternal blood, fetal appendages, placenta, umbilical cord, and umbilical vein blood, although their detection alone does not establish maternal–fetal transfer of PU particles [149,151]. A preprint reported higher PU mass concentrations in preterm than in term placentae; this finding remains preliminary [150].

Findings from reproductive and urinary samples provide further evidence of PU occurrence. PU-containing particles have been detected in human endometrium and urine [153,160]. A conference abstract additionally reported PU in follicular and seminal fluids [152]. Another conference abstract reported an association between higher PU mass concentrations in follicular fluid and lower fertilization rates during in vitro fertilization (IVF) [154].

PU has been reported in sputum, BALF, and other respiratory samples collected from never-smokers, smokers, and patients with respiratory diseases [85,86,87,88,89,90]. Particles identified as polyurethane glycol were also reported in middle-ear effusion fluid from pediatric patients [91]. In a larger BALF study, MPs were detected in 96.26% of participants, with PE, PU, and PVC among the most prevalent polymers [86].

Occupational and high-exposure settings may create additional exposure scenarios. In a cross-sectional study from Chengdu, participants from an area near a plastic factory were compared with a lower-exposure group from an urban park area. PU was the dominant polymer in the high-exposure environment, accounting for 36.36% of MPs in soil and 38.05% of MPs in air. The same polymer also represented the largest fraction in intestinal secretions from the high-exposure group, where total MP levels were higher than in the lower-exposure group. These differences were accompanied by changes in nasal and intestinal microbiota, suggesting that high environmental MP exposure may be reflected not only in particle burden but also in mucosal microbial communities [155].

Human tissue studies provide additional evidence of PU occurrence in vascular and other tissue samples. Pyrolysis–gas chromatography/mass spectrometry (Py-GC/MS) analysis showed higher total measured polymer concentrations in femoral atherosclerotic plaques than in non-diseased arteries, with higher PU concentrations in plaques from patients with chronic limb-threatening ischemia than in those with claudication [156]. However, this analysis did not distinguish MPs from NPs. PU was also identified among the polymers detected in paired breast tumor and para-tumor tissues [157]. Studies of human blood and carotid plaques, as well as fibrotic intestinal tissue and mesenteric adipose tissue from patients with Crohn’s disease, further show that MPs have been detected in these human biological matrices [158,159].

The human biomonitoring findings summarized in Table 1 document PU occurrence in biological samples, with study-specific quantitative data and contamination-control information provided where reported. These findings provide a basis for investigating the potential health implications of PU exposure. Establishing whether PU contributes to the observed health outcomes requires further evidence linking exposure to biological effects. Where PU was identified alongside other polymers, associations involving total microplastic burden cannot be attributed specifically to PU. Polymer-specific measurements and associations are therefore distinguished from findings concerning the overall mixed-polymer burden.

### 5.2. In Vitro Evidence

In vitro evidence on PU-derived or PU-related materials is still limited. Available cell-based data mainly concern selected exposure forms, including PU-derived DOM, chlorinated TPU leachates, and particles released from urethane-based orthodontic materials [35,50,72]. These models show that PU-related biological responses depend not only on the polymer itself, but also on the tested exposure form and cell type. The available studies provide formulation-specific insights, while differences in material composition, particle size, chemical release, dose, and biological model limit comparisons across the PU family.

Studies of DOM and leachates from aged or chemically transformed PU/TPU provide evidence of biological activity associated with the released chemical fraction [35,50]. The amount of DOM released from UV-aged PU MPs increased with aging time and was associated with higher cytotoxicity toward human colon adenocarcinoma cells [35]. Similarly, UV-aged TPU exposed to chlorination released more organic compounds and formed disinfection by-products, while leachates from chlorinated, UV-aged TPU were more cytotoxic toward human hepatocarcinoma cells than leachates from virgin TPU [50]. A broader comparison of consumer-product extracts identified high in vitro toxicity for PU and PVC, low or no toxicity for high-density PE, and variable responses for low-density PE and PP [161]. These findings highlight differences in the biological activity of extractable chemicals, while their relevance to the relative toxicity of PU, PE, PP, and PVC particles remains uncertain.

Evidence from direct exposure to solid TPU particles should be considered separately from the DOM and leachate studies. A conference abstract reported no initial cytotoxicity of solvent-precipitated TPU particles in Calu-3 cells (a human bronchial epithelial cell line) at concentrations up to 1 mg/mL; this result should be considered preliminary [162]. A later characterization study confirmed that solvent precipitation can generate respirable TPU test materials and emphasized the importance of particle size, composition, surface properties, impurities, endotoxin content, and solvent residues when preparing microplastics for inhalation studies [163]. Digestive models relevant to human exposure provide additional context because the bioaccessibility or desorption of microplastic-associated xenobiotics, including compounds associated with TPU, may change in simulated gastric and intestinal fluids [164,165].

Particles released from PU-related oral materials have also been tested in macrophage models. Orthodontic polymers, including urethane-based direct-printed materials, released MPs and NPs in artificial saliva, and particles of approximately 2–10 μm were readily phagocytosed by macrophages. Exposure to urethane-based direct-printed materials was also associated with a shift toward inducible nitric oxide synthase (iNOS)^+^/cluster of differentiation 86 (CD86)^+^ M1-like pro-inflammatory macrophage phenotypes [72]. These findings provide insights into the biological effects of the tested orthodontic formulations, with their relevance to other PU/TPU particles depending on material composition and particle characteristics [72]. The observed responses reflect exposure to the released particle mixtures, while the specific contribution of the NP fraction remains to be clarified [72].

Evidence on PU-specific effects at epithelial barriers is still limited, but the available models suggest several directions for further research. PU-derived DOM was toxic to intestinal epithelial-like cells, while endometrial organoid, Calu-3, and air–liquid interface (ALI) models show how epithelial systems may be used to study particle–barrier interactions after ingestion or inhalation [35,153,162,163]. Fluorescent PU microspheres may also support this work by allowing PU particle uptake and distribution to be tracked in cells and animal models [166]. At present, epithelial transport, barrier disruption, and chronic remodeling remain plausible but insufficiently defined PU-specific outcomes.

### 5.3. In Vivo Evidence

PU-specific in vivo evidence is still sparse and comes mainly from aquatic or non-mammalian models. In adult zebrafish, fluorescent PU-MPs were ingested and detected in the gills and gastrointestinal tract. Exposure was associated with gill damage, including necrosis and changes in secondary lamellae, as well as gastrointestinal and liver alterations such as epithelial detachment, bowel-wall thinning, inflammation, and tissue injury [18].

In larval fathead minnows, exposure to PU particles, PU-associated tris(1-chloro-2-propyl) phosphate (TCPP) leachate, or their combination reduced growth at 12 days post-hatch, and TCPP uptake was detected in exposed larvae [19]. In zebrafish sac fry, a comparison of commercial microplastics showed that PU reduced body length and pericardial sac size at both 1 and 10 μg/mL, whereas PVC reduced body length at 10 μg/mL. PP increased pericardial sac size at both concentrations, while high-density PE increased pericardial sac size at 10 μg/mL [20]. These endpoint-dependent responses highlight developmental effects across several polymers without establishing PU as consistently more toxic.

Beyond aquatic models, a few studies on PU-based materials show how these materials can affect living tissues under different exposure conditions. In earthworms, castor-oil-based waterborne PU caused charge-dependent toxicity, including impaired growth and reproduction, oxidative stress, histopathological damage, lysosomal injury, deoxyribonucleic acid (DNA) damage, apoptosis, and transcriptomic changes related to detoxification and cell-death pathways [167]. In a biomedical implantation model, degradation of PU-containing vascular grafts was accompanied by a foreign-body response involving macrophages, epithelioid cells, multinucleated giant cells, fibroblasts, and capillaries within the degrading graft lattice [168].

The in vivo findings should be interpreted in the context of their respective exposure conditions. Studies involving PU particles, TCPP leachate, and combined exposure address distinct test conditions [18,19,20]. Findings from waterborne PU formulations and implanted PU-containing grafts provide additional material-specific context but are not equivalent to evidence from exposure to environmental PU microplastics or nanoplastics [167,168]. These different forms of evidence remain insufficient to establish PU-specific toxicity in humans.

Selected experimental studies are compared in Table 2, highlighting differences in material characteristics, dose, exposure duration, biological models, and outcomes, while distinguishing particle exposure from exposure to dissolved fractions and leachates.

## 6. One Health Perspective

A One Health perspective links the occurrence of PU-containing particles across environmental compartments and organisms with potential human exposure, while distinguishing evidence on MPs from the more limited evidence on NPs.

### 6.1. Soil, Microbial Communities and Lower Trophic Levels

Terrestrial and soil-related studies show that PU-containing particles may interact with living systems before they enter food-related or human-relevant exposure pathways. MPs derived from PU-containing coated fertilizers and PU films have been examined in soil–crop systems, including tomato, rice, and broader agricultural settings. Reported effects include changes in seedling growth, soil enzyme activity, cadmium availability, microbial colonization, and plant metabolic pathways [135,136,138,139,141]. In commercial organic fertilizers from China, PU was among the dominant polymer types, and particles below 100 μm accounted for most detected MPs [169]. Effects on the earthworm Eisenia fetida have also been observed, supporting the ecological relevance of these findings [167].

Microbiological and lower-trophic-level studies broaden this ecosystem perspective. In salt-marsh sediment microcosms, PU foam MPs changed sediment microbial communities and promoted nitrification and denitrification, linking PU-containing particles with changes in nitrogen-cycling processes [170]. In the deep sea, PU and PS substrates deployed for 420 days at a depth of 1796 m in the Rockall Trough were colonized by bacterial communities, demonstrating PU’s role as a substrate in the deep-sea plastisphere [171]. Bioindicator studies add another ecological context. For example, in lichens from Yozgat Çamlık National Park in Türkiye, PU represented 42% of the identified anthropogenic microfibers [172].

### 6.2. Aquatic Organisms, Wildlife and Sensitive Habitats

Aquatic studies extend the relevance of PU-containing MPs beyond dietary exposure by placing them within benthic, pelagic, and trophic interactions. PU has been identified in organisms occupying different ecological niches, including the benthic deposit-feeder Holothuria scabra and selected freshwater and marine taxa [99,100,101,102,103,104,105,106,173,174]. In the Laccadive Sea, MP abundance increased with trophic level, while high-density polymers, including PU, became more prominent in bathypelagic organisms, providing evidence relevant to trophic transfer [174]. Commercial aquaculture feeds may further introduce PU-containing MPs into farmed aquatic systems [137].

Wildlife studies add evidence from higher trophic levels. In sea turtles, PU has been identified among ingested polymer types, although usually as a minor fraction and often in debris larger than the conventional MNP size range [175,176]. In juvenile green sea turtles, plastic contamination was detected in fecal samples from 98% of individuals, and PU was among the polymers identified [177]. Birds also provide useful ecological evidence. In regurgitated pellets from coexisting raptors, MPs were detected in 92% of common kestrel pellets and 83% of barn owl pellets, with PU representing 9.2% of the identified polymers [178]. In spotted seals from the Bohai Sea, small MPs were detected in fecal samples. Although PU was not reported as the dominant polymer, it contributed to the calculated polymer hazard index [179].

Coral-associated habitats indicate that PU-containing particles may also reach sensitive marine environments. In a cold-water coral reef protected area in the Sea of the Hebrides, benthic macrofauna ingested microparticles, and PU was identified among the polymer types [180]. In a protected Mediterranean Cladocora caespitosa coral bed, high concentrations of MPs and microrubber were reported in sediments, with PU contributing to the polymer profile [181]. Coral-associated habitats may therefore act as retention zones for small particles, where exposure of benthic organisms depends not only on sediment contamination but also on habitat structure and feeding behavior.

These examples place PU-derived MNPs within a broader environmental continuum that includes soils, microbial communities, aquatic organisms, wildlife, protected habitats, food webs, and human-relevant exposure routes. Of particular relevance is the occurrence of PU-containing particles across compartments that are often examined separately, including soils, sediments, organisms, food systems, and human biological samples. Integrating environmental monitoring, wildlife biomonitoring, trophic-transfer studies, food-system analysis, and human biomonitoring would help clarify the ecological and human-health relevance of PU-derived particles.

## 7. Knowledge Gaps and Future Directions

Although evidence of human exposure to PU-containing MPs is increasing, substantial knowledge gaps remain regarding PU nanoplastic exposure, particle fate, and health effects.

### 7.1. Limited Clinical Evidence

PU occurrence is increasingly reported in human samples, predominantly as MPs or as polymer mass without size resolution, but its clinical significance remains uncertain because most studies assess mixed-polymer exposure rather than PU alone. Dose–response relationships, tissue kinetics, and disease-specific mechanisms remain insufficiently characterized, consistent with limitations identified in broader reproductive-health reviews [182]. Future studies should determine whether PU burden, particle size, polymer subtype, weathering state, additive profile, or tissue localization is associated with inflammation, barrier dysfunction, microbiome changes, reproductive outcomes, vascular pathology, respiratory disease, or other clinically meaningful endpoints [85,86,87,88,89,90,91,108,147,148,149,150,151,152,153,154,155,156,157,158,159,160,182,183,184].

### 7.2. Analytical Limitations in PU Detection

Evidence is currently stronger for PU occurrence and experimental biological effects than for PU-specific health outcomes in humans [18,19,85]. Associations observed in human studies warrant further investigation. Causal interpretation requires consideration of study quality, potential confounding, exposure timing, and the relevance of the exposure dose.

Analytical uncertainty remains one of the central barriers in PU research. PU is a broad family of materials that vary in their diisocyanates, polyols, chain extenders, additives, and production processes. This diversity makes PU identification challenging in human tissues, sputum, BALF, urine, food, wastewater, sludge, sediments, dust, and soil. Pyrolysis-based approaches indicate that reliable PU identification may require subclass-specific markers, including methylene diphenyl diisocyanate (MDI)- and toluene diisocyanate (TDI)-based markers, and that derivatization or thermochemolysis can improve detection and reduce matrix interference [185,186,187,188]. Sample preparation is equally important because digestion protocols may damage or eliminate PU particles. For example, nitric acid caused at least 90% mass loss for several polymers, including polyurethane, while strong acids or high temperatures may degrade or dissolve PU and other polymers [189,190].

The choice of analytical method also determines what can be concluded from a sample. Among the biomonitoring studies summarized in Table 1, sputum analysis identified PU using LDIR with supplementary micro-Fourier-transform infrared spectroscopy (μFTIR) [85]. In urine, PU identification was based on LDIR, whereas the accompanying Py-GC/MS analysis reported PE, PVC, and polyamide 66 (PA66) [160]. Studies of femoral plaques and paired blood/carotid plaque samples instead used Py-GC/MS to quantify PU mass [156,158]. These examples illustrate how the analytical approach determines whether PU occurrence is described through identified particles or polymer mass. μFTIR, Raman spectroscopy, LDIR, Py-GC/MS, pyrolysis–gas chromatography–ion mobility mass spectrometry (Py-GC-IM-MS), nuclear magnetic resonance (NMR)-based methods, fluorescence staining, and multimodal workflows can all support PU analysis, but they differ in size limits, spectral-library dependence, mass quantification, morphology preservation, and matrix compatibility [76,84,133,185,186,187,188,189,190,191,192,193,194,195,196,197,198,199,200]. Future studies therefore require validated matrix-specific protocols, procedural blanks, recovery tests, reference materials, harmonized detection limits, and a clear distinction between particle-count and mass-based data [69,76,84,133,148,185,189,190,191,192,193,194,195,196,197,198,199,200]. A key priority is the development of well-characterized PU reference materials representing different formulations and particle sizes, together with interlaboratory comparisons and external quality assessment schemes for human biomonitoring. These tools would help evaluate recovery, polymer identification, and quantitative agreement across laboratories, strengthening confidence in PU measurements in complex biological samples.

### 7.3. MPs Versus NPs

Human biomonitoring evidence for PU predominantly concerns MPs and reflects the particle-size ranges accessible to the analytical methods used. When comparing studies, it is helpful to distinguish the size boundaries used to define MPs and NPs from method-specific detection limits and the smallest particles observed in individual samples. For example, studies identifying PU among other polymers reported predominantly 20–50 μm particles in mother–infant samples and 20–100 μm particles in maternal blood, fetal appendages, and umbilical vein blood, while sputum analysis covered a 20–500 μm range [85,108,147,149].

These size distributions describe the detected polymer mixtures and should not be interpreted as PU-specific distributions. Other studies quantified PU by Py-GC/MS without resolving particle size; such measurements do not establish the presence or relative contribution of a nanoplastic fraction [150,154,156,158].

Experimental studies nevertheless demonstrate the release of both micro- and nanoscale particles from selected PU-containing materials and TPU during aging [25,27,40,41,51]. During aging, fragmentation can generate nanoscale fragments from initially micrometer-sized PU particles, so the resulting material may span both size categories [40,41]. These operational categories help describe exposure, but do not, by themselves, define distinct biological responses; uptake and effects need to be interpreted using the particle sizes actually tested. This evidence supports the potential formation of PU-related NPs but does not establish their occurrence, internal dose, or health effects in humans. Findings on PU MPs should therefore not be extrapolated to PU NPs without size-specific evidence, and biological responses to mixtures containing both fractions should not be attributed to NPs alone.

### 7.4. Need for Human-Focused Mechanistic Research

Future research should move beyond descriptive particle detection and focus on human-relevant mechanistic studies linking exposure to PU-derived MNPs with biological responses. Priority should be given to standardized exposure scenarios, well-characterized and environmentally relevant PU particles, aged materials, harmonized dose metrics, and experimental models representing the respiratory, intestinal, placental, reproductive, and vascular systems (the main research priorities and methodological needs are summarized in Table 3) [18,19,25,26,35,40,41,50,51,72,162,163,166]. Addressing these gaps will improve understanding of PU-specific mechanisms and support more reliable human health-risk assessment.

## 8. Conclusions

Available evidence indicates that PU-containing materials can release particles and dissolved chemicals during use and aging, and PU has been identified in human biological samples. PU formulation and product structure influence environmental transformation and chemical release, making material-specific characterization central to interpreting exposure and biological effects. Medical and occupational settings deserve particular attention alongside environmental exposure pathways. Experimental studies provide evidence of tissue damage and developmental changes in fish exposed to PU particles, cytotoxicity of selected PU-derived dissolved fractions and leachates, and altered macrophage responses to certain urethane-containing orthodontic formulations. These findings provide insights into potential biological effects and should be interpreted in the context of the material, exposure form, dose, and model tested. They may help advance understanding of human health effects, although causal links between PU particle exposure and disease remain to be established.

Human biomonitoring currently provides more information on micrometer-sized particles and polymer mass than on PU NPs. Laboratory aging studies demonstrate the formation of nanoscale fragments, while their occurrence, internal dose, and health implications in humans require further investigation. Well-characterized PU materials, validated analytical methods, appropriate contamination controls, and studies reflecting human exposure conditions would strengthen the evidence base and support more reliable assessment of potential health risks.

## Figures and Tables

**Table 1:** Human biomonitoring evidence for PU in biological samples.

Biological Sample	Study Characteristics *	PU-Specific Findings	Contamination Controls	Ref.
Sputum	*n* = 22 hospitalized patients with respiratory diseases; LDIR (analytical size range: 20–500 μm), with supplementary μFTIR.	PU accounted for 33.95% of identified MPs; median PU concentration: 8 particles/10 mL sputum (Q1–Q3: 3.25–10.75).	One procedural blank (75% ethanol); polymer-specific blank correction; PU recovery: 84% in a soil-surrogate-matrix test.	[85]
	*n* = 30; optical microscopy: 20–500 μm; μRaman identification of 30 selected sputum particles > 50 μm.	PU: 18/30 particles selected for μRaman analysis; all PU particles were fibers.	Procedural blank: 12 fibrous particles/10 mL; size-specific blank correction; recovery: NR.	[87]
BALF	*n* = 454; LDIR (analytical size range: 20–500 μm), with supplementary SEM.	PU detected in 48.68% of BALF samples; median 0.00 particles/g (Q1–Q3: 0.00–9.79).	Forty saline procedural controls; control PU: median 0.00 particles/g (IQR 0.25). Blank correction and PU recovery: NR.	[86]
	*n* = 32 participants: 17 smokers and 15 nonsmokers; LDIR (analytical size range: 20–500 μm).	Blank-corrected PU (mean ± SD): smokers, 10.06 ± 16.30 particles/g; nonsmokers, 4.03 ± 7.67 particles/g.	Six procedural blanks; blank subtraction and blank-based LOD/LOQ assessment. PU recovery: NR.	[88]
	*n* = 18 patients with suspected ILD and 7 controls; μRaman; observed MPs: 4.19–792 μm.	Three PU particles detected in BALF from three ILD patients (40.48–98.03 μm); 20.0% of MPs identified in BALF.	Laboratory and bronchoscopy-room controls; no PU listed in control samples. Control-associated particles excluded; PU recovery: NR.	[89]
	*n* = 18 never-smokers; LDIR (analytical size range: 20–500 μm), with supplementary SEM.	PU: 0.16 particles/g (median; IQR 0.24); 1.4% of detected MPs.	Six saline procedural controls; control PU: median 0 particles/g (IQR 0.07). Recovery: PS 92%, PP 89%, PE 78%; PU recovery and blank correction: NR.	[90]
Urine	*n* = 12 participants (12 urine samples); PU identified by LDIR (20–500 μm). Py-GC/MS did not target PU.	PU detected in 2/12 urine samples, at 13.28 and 20.77 particles/kg.	Ultrapure-water blanks and controls covering collection air, wipes, and containers: no MNPs detected by LDIR or Py-GC/MS; results reported without blank correction. Blank number and PU recovery: NR.	[160]
Placenta/ meconium	18 placental and 12 meconium samples; LDIR; analytical size range: 20–500 μm.	PU, median (Q1–Q3): placenta, 5.5 (3.0–9.7) particles/g; meconium, 10.7 (3.6–18.7) particles/g.	Three procedural blanks; particles in blanks did not meet the spectral identification criterion. PU recovery: 84% in a soil surrogate matrix. Blank correction: NR.	[108]
	18 placental and 12 meconium samples from the same cohort as [108]; LDIR; analytical size range: 20–500 μm.	PU, median (Q1–Q3): placenta, 5.5 (3.0–9.7) particles/g; meconium, 10.7 (3.6–18.7) particles/g. PU proportions: 28.75% and 22.58%, respectively.	Three procedural blanks; no particles met the polymer-identification threshold. PU recovery: 84% in a soil surrogate matrix. Blank correction: NR.	[147]
	*n* = 2 mother–infant pairs following cesarean delivery; placental tissue and neonatal meconium; μFTIR (analytical size range: 50–500 μm); pilot study.	PU detected in a central placental sample; PU-specific particle count/concentration: NR.	Procedural, instrument/container, and airborne controls; PU detected in operating-room airborne fallout; blank correction and numerical recovery: NR.	[148]
Endometrium/ reproductive fluids	Follicular and seminal fluids; *n* = 25 women undergoing follicular aspiration and 18 men undergoing semen analysis; LDIR; conference abstract.	PU reported in follicular and seminal fluids; detected in >50% of follicular-fluid samples; PU concentrations: NR in the conference abstract.	Collection/storage containers tested for MP contamination; procedural blanks, blank correction, and recovery: NR in the conference abstract.	[152]
	Endometrium; *n* = 22; μRaman and FTIR imaging; Raman-listed particle diameters: 1.95–12.05 μm.	PU: approximately 17% of MPs identified by infrared imaging (estimated from panel D of Figure 2 in the cited study).	Milli-Q water procedural blanks; system blanks negative; no blank correction applied. PU recovery: NR.	[153]
	Follicular fluid; *n* = 33 follicular-fluid samples from independent IVF cycles; Py-GC/MS; particle size not resolved; conference abstract.	PU: 0.19 ± 0.00 μg/mL (mean ± SD, as reported).	Technical background controls; blank type, blank correction, and recovery: NR in the conference abstract.	[154]
Blood/ vascular plaques/tumor and inflammatory tissues	Paired blood samples collected before and immediately after PCI; *n* = 23 patients; LDIR (particles > 20 μm).	PU: 0.61 ± 1.20 before PCI and 10.70 ± 4.58 particles/10 mL blood after PCI (mean ± SD).	Six blood-procedure blanks: no MPs detected; no blank correction applied. PU recovery: 89.4% (RSD 6.8%; three replicate tests).	[31]
	Femoral plaques and control carotid arteries; 10 femoral plaques from 8 patients; 7 age-matched control carotid arteries; Py-GC/MS; particle size not resolved.	Femoral plaque PU concentrations: 17.4 ± 12.1 μg/g in patients with chronic limb-threatening ischemia versus 1.69 ± 2.9 μg/g in patients with claudication.	Containers, pipette tips, and centrifuge tubes screened; formalin and KOH blanks analyzed. No detectable polymers in the processed KOH blank. Blank correction and PU recovery: NR.	[156]
	Paired breast tumor and para-tumor tissues; *n* = 7 patients (14 samples; 7 pairs); LDIR (20–500 μm), with supplementary SEM.	PU accounted for 16.5% of MPs in tumor tissues and 6.8% in para-tumor tissues.	Three procedural blanks; mean PU background: 0.67 particles/blank. Results corrected using polymer- and size-specific blank values. PU recovery: NR.	[157]
	Paired blood and carotid plaque samples from 20 patients; Py-GC/MS; particle size not resolved.	PU accounted for 0.50% of total measured polymer mass in blood and 0.60% in carotid plaques.	Two field blanks and two procedural blanks; all polymers below method detection limits. Blank correction and PU recovery: NR.	[158]
	Ileal and mesenteric adipose tissues; *n* = 10 patients with Crohn’s disease (32 samples); LDIR (analytical size range: 20–500 μm).	PU proportions among identified MPs: 6.91% in uninvolved ileum, 21.60% in involved ileum, 2.85% in adjacent uninvolved mesenteric adipose tissue, and 15.96% in creeping fat.	Procedural Milli-Q water blanks: no MPs detected. Blank number, numerical blank correction, and PU recovery: NR.	[159]

Abbreviations: BALF = bronchoalveolar lavage fluid; FTIR = Fourier-transform infrared spectroscopy; ILD = interstitial lung disease; IQR = interquartile range; IVF = in vitro fertilization; KOH = potassium hydroxide; LDIR = laser direct infrared spectroscopy; LOD = limit of detection; LOQ = limit of quantification; MNPs = micro- and nanoplastics; MPs = microplastics; *n* = sample size (participants or samples, as specified); NR = not reported; PCI = percutaneous coronary intervention; PE = polyethylene; PP = polypropylene; PS = polystyrene; PU = polyurethane; Py-GC/MS = pyrolysis–gas chromatography/mass spectrometry; Q1–Q3 = first and third quartiles; ref. = references; RSD = relative standard deviation; SD = standard deviation; SEM = scanning electron microscopy; μFTIR = micro-Fourier-transform infrared spectroscopy; μRaman = Raman microspectroscopy. * Analytical methods and particle-size ranges are provided where verified. Reported analytical ranges are distinguished from observed particle sizes. Unless specified as PU-specific, size ranges refer to the overall detected particle mixture. NR indicates that the information was not reported in the cited source; it does not imply that the procedure was not performed.

**Table 2:** Selected toxicological studies of PU-related particles and chemical fractions.

Material, Size and Treatment	Dose and Duration	Biological Model	Main Outcome	Exposure Type	Ref.
Ground rigid PU foam; <100, 100–300 and 300–1000 μm; no aging described	0, 1, 10, 100 and 1000 μg/L; 10 days	Adult zebrafish	Size- and concentration-dependent mortality; gill, intestinal and liver damage.	Particles	[18]
Mechanically fragmented PU, 100–500 μm, with/without TCPP; separate leachate treatment	20,000 particles/L (~24 mg/L); 28 days	Larval fathead minnow	Reduced growth at day 12, but not day 28; TCPP uptake in chemical-containing treatments.	Particles, leachate and combined exposure	[19]
Virgin/pure PU, mechanically processed and passed through a 2 mm sieve; size distribution not reported	1 and 10 μg/mL; 3–96 hpf	Zebrafish embryos/larvae	Reduced body length and pericardial sac size.	Particles	[20]
Solvent-precipitated TPU; 95% of particles < 10 μm	Up to 1 mg/mL; duration not specified in the conference abstract	Calu-3 cells	No cytotoxicity detected by WST assay.	Particles	[162]
DOM from UV-aged PU MPs; parent material passed through a 200-mesh screen	PU-derived DOM; final exposure concentration not specified; 24 and 48 h	Human colonic adenocarcinoma cells (Caco-2)	Cytotoxicity increased with parent-material aging.	Dissolved fraction	[35]
Leachates from chlorinated virgin and UV-aged TPU; parent particles: 75–150 μm	Serial dilutions of concentrated leachate extracts; 48 h	Human hepatocarcinoma cells (HepG2)	Approximately 10-fold higher cytotoxicity of leachates from chlorinated UV-aged TPU versus chlorinated virgin TPU.	Leachates	[50]
Orthodontic formulations, including PU and urethane-based materials; ~10 nm–100 μm across formulations; 1 week in artificial saliva with daily agitation	1000 particles/ co-culture; 3 days	Murine bone marrow-derived macrophages	Material-dependent M1-like differentiation; directly observed uptake of 2–10 μm particles.	Mixed-size particles	[72]

Note: Doses are reported in their original units. Comparisons between mass-based and particle-number concentrations require information on particle size, shape, and density. Material preparation and aging may influence particle properties and chemical release. The relevance of laboratory effect concentrations to human health risk requires consideration of exposure conditions and available PU-specific exposure data. Abbreviations: DOM = dissolved organic matter; hpf = hours post-fertilization; M1 = classically activated pro-inflammatory macrophage phenotype; MPs = microplastics; PU = polyurethane; TCPP = tris(1-chloro-2-propyl) phosphate; TPU = thermoplastic polyurethane; UV = ultraviolet; WST = water-soluble tetrazolium.

**Table 3:** Key research priorities and future directions for research on PU-derived MNPs.

Human-Relevant Question	Suggested Experimental Approach	PU-Relevant Test Material	Priority Endpoints	Supporting Evidence
Can inhaled PU-derived particles interact with the respiratory barrier?	ALI airway models using Calu-3 or primary airway epithelial cells, preferably with macrophages	Respirable PU/TPU particles, including <10 μm and aged fractions	deposition, epithelial uptake, barrier integrity, oxidative stress, cytokine release, macrophage interaction	[86,87,88,89,90,162,163]
Can urethane-based dental materials activate immune cells?	Artificial-saliva aging followed by macrophage and oral epithelial-cell exposure	Aligner-derived particles and urethane-based printed dental materials	particle release, phagocytosis, macrophage polarization, inflammatory cytokines, oral epithelial response	[29,72]
Does gastrointestinal exposure depend on particles or released chemicals?	Intestinal epithelial models combined with simulated gastric and intestinal fluids	Aged PU/TPU particles, PU-derived DOM, leachates, and contaminant-loaded particles	cytotoxicity, ROS formation, barrier disruption, xenobiotic bioaccessibility, chemical desorption	[35,50,164,165]
Can PU-containing particles affect reproductive or maternal–fetal interfaces?	Endometrial organoids, placental barrier models, and follicular-cell or reproductive-fluid models	PU-containing mixed MPs, aged PU particles, and reference particles	uptake, translocation, apoptosis, inflammation, organoid growth, fertilization- or implantation-related endpoints	[108,147,148,149,150,151,152,153,154]
Are medical procedures a source of internal PU exposure?	Simulated-use studies of medical devices combined with blood-contact models	Particles released from disposable devices, IV admixtures, hemodialysis systems, and interventional devices	particle release into fluids or blood, endothelial activation, immune-cell uptake, coagulation markers, inflammation	[30,31,32,71]
How does aging change the biological activity of PU?	Parallel testing of pristine, UV-aged, chlorinated, ozonated, and weathered PU/TPU particles and leachates	PU/TPU particles exposed to UV, chlorination, ozonation, salinity, or mechanical aging	surface oxidation, DOM release, additive release, disinfection by-products, ROS generation, cytotoxicity	[33,34,35,50,64,122,123,124]
How can PU particles be tracked in biological systems?	Imaging-based uptake studies using chemically validated reference particles	Fluorescent PU microspheres and well-characterized PU reference materials	cellular uptake, intracellular localization, biodistribution, persistence, particle/leachate distinction	[18,166]

Abbreviations: ALI = air–liquid interface; DOM = dissolved organic matter; IV = intravenous; MNPs = micro- and nanoplastics; MPs = microplastics; PU = polyurethane; ROS = reactive oxygen species; TPU = thermoplastic polyurethane; UV = ultraviolet.

## Data Availability

No new data were created or analyzed in this study. Data sharing is not applicable to this article.

## Abbreviations

The following abbreviations are used in this manuscript:

ABS	acrylonitrile butadiene styrene
ALI	air–liquid interface
APC	article processing charge
BALF	bronchoalveolar lavage fluid
CD86	cluster of differentiation 86
DNA	deoxyribonucleic acid
DOM	dissolved organic matter
EAACI	European Academy of Allergy and Clinical Immunology
FTIR	Fourier-transform infrared spectroscopy
HepG2	human hepatocarcinoma cells
hpf	hours post-fertilization
ILD	interstitial lung disease
iNOS	inducible nitric oxide synthase
IQR	interquartile range
IV	intravenous
IVF	in vitro fertilization
KOH	potassium hydroxide
LDIR	laser direct infrared spectroscopy
LOD	limit of detection
LOQ	limit of quantification
M1	classically activated/pro-inflammatory macrophage phenotype
MDI	methylene diphenyl diisocyanate
MEDLINE	Medical Literature Analysis and Retrieval System Online
MNPs	micro- and nanoplastics
MPs	microplastics
NMR	nuclear magnetic resonance
NPs	nanoplastics
NR	not reported
PA	polyamide
PA66	polyamide 66
PC	polycarbonate
PCI	percutaneous coronary intervention
PE	polyethylene
PE-HD/MD	high- and medium-density polyethylene
PE-LD/LLD	low-density and linear low-density polyethylene
PET	polyethylene terephthalate
PMMA	poly(methyl methacrylate)
PP	polypropylene
PS	polystyrene
PU	polyurethane
PU-MPs	polyurethane microplastics
PVC	polyvinyl chloride
Py-GC-IM-MS	pyrolysis–gas chromatography–ion mobility mass spectrometry
Py-GC/MS	pyrolysis–gas chromatography/mass spectrometry
Q1–Q3	first–third quartiles
ROS	reactive oxygen species
RSD	relative standard deviation
SD	standard deviation
SEM	scanning electron microscopy
TCPP	tris(1-chloro-2-propyl) phosphate
TDI	toluene diisocyanate
TPU	thermoplastic polyurethane
UV	ultraviolet
WST	water-soluble tetrazolium
μFTIR	micro-Fourier-transform infrared spectroscopy
μRaman	Raman microspectroscopy
